# Hepatocellular carcinoma immune prognosis score predicts the clinical outcomes of hepatocellular carcinoma patients receiving immune checkpoint inhibitors

**DOI:** 10.1186/s12885-023-11678-5

**Published:** 2023-12-01

**Authors:** Rujia Zhang, Haoran Zhao, Peng Wang, Zuoming Guo, Chunxun Liu, Zhaowei Qu

**Affiliations:** 1 Department of Operating Room, The Second Affiliated Hospital of Harbin Medical University, Harbin Medical University, Harbin, 150086 Heilongjiang China; 2 Department of Hepatobiliary and Pancreatic Surgery, Harbin Medical University Cancer Hospital, Harbin Medical University, Harbin, 150081 Heilongjiang China

**Keywords:** Prognosis score, Hepatocellular carcinoma, Immune checkpoint inhibitors, Clinical outcomes, Non-invasive biomarkers

## Abstract

**Objective:**

The predictive biomarkers of immune checkpoint inhibitors (ICIs) in hepatocellular carcinoma (HCC) still need to be further explored. This study aims to establish a new immune prognosis biomarker to predict the clinical outcomes of hepatocellular carcinoma patients receiving immune checkpoint inhibitors.

**Methods:**

The subjects of this study were 151 HCC patients receiving ICIs at Harbin Medical University Cancer Hospital from January 2018 to December 2021. This study collected a wide range of blood parameters from patients before treatment and used Cox’s regression analysis to identify independent prognostic factors in blood parameters, as well as their β coefficient. The hepatocellular carcinoma immune prognosis score (HCIPS) was established through Lasso regression analysis and COX multivariate analysis. The cut-off value of HCIPS was calculated from the receiver operating characteristic (ROC) curve. Finally, the prognostic value of HCIPS was validated through survival analysis, stratified analyses, and nomograms.

**Results:**

HCIPS was composed of albumin (ALB) and thrombin time (TT), with a cut-off value of 0.64. There were 56 patients with HCIPS < 0.64 and 95 patients with HCIPS ≥ 0.64, patients with low HCIPS were significantly related to shorter progression-free survival (PFS) (13.10 months vs. 1.63 months, *P* < 0.001) and overall survival (OS) (14.83 months vs. 25.43 months, *P* < 0.001). HCIPS has also been found to be an independent prognostic factor in this study. In addition, the stratified analysis found a significant correlation between low HCIPS and shorter OS in patients with tumor size ≥ 5 cm (*P* of interaction = 0.032). The C-index and 95% CI of the nomograms for PFS and OS were 0.730 (0.680–0.779) and 0.758 (0.711–0.804), respectively.

**Conclusions:**

As a new score established based on HCC patients receiving ICIs, HCIPS was significantly correlated with clinical outcomes in patients with ICIs and might serve as a new biomarker to predict HCC patients who cloud benefit from ICIs.

## Introduction

Hepatocellular carcinoma (HCC) is the sixth most common cancer in the world, with a high incidence rate and mortality, especially in Asian countries [1]. Surgery was the main treatment method for HCC, but patients have a lower surgical resection rate and a higher risk of postoperative recurrence [2, 3]. In addition, HCC was equally insensitive to radiotherapy and chemotherapy, resulting in fewer treatment strategies for patients to choose from [4]. Some patients who were unable to receive surgery or had postoperative recurrence have to receive conservative treatment.

The emergence of immune checkpoint inhibitors (ICIs) has brought new hope to many patients with refractory cancer, including HCC patients [5–7]. However, the low response rate of ICIs in solid tumors remained a significant obstacle to their promotion. Some clinical trials have found that although ICIs could prolong the survival of HCC patients, their objective efficacy rate was only about 20%, still at a relatively low level [8–11]. To improve the response rate of HCC patients, people have begun to focus on the study of biomarkers for ICIs. Existing biomarkers such as PD-L1 expression levels and Combined Positive Score (CPS) have been widely used in clinical practice [12–14]. However, they still could not cover all patients, some studies have found that patients with lower levels of PD-L1 expression and CPS could still benefit from ICIs [15, 16]. In addition, due to the low surgical and biopsy rates of HCC, the use of biomarkers based on pathological detection was further restricted. Therefore, it was important to explore non-invasive biomarkers that could be applied to HCC patients receiving ICIs.

Several classic biomarkers that reflect a patient’s inflammation and nutritional status, including the prognostic nutritional index (PNI), systemic immune-inflammation index (SII), and advanced lung cancer inflammation index (ALI), have been found to be related to the efficacy of ICIs in various cancers [17–20]. Their prognostic value in HCC patients receiving ICIs has also gradually been confirmed [21, 22]. However, the mechanism of ICIs differed significantly from classic treatment methods such as surgery, chemotherapy, and targeted therapy, and the immune characteristics of HCC patients was also different from other tumors. Some new biomarkers established based on HCC patients, such as CRAFITY, have shown tremendous potential in predicting the effectiveness of immunotherapy [23, 24]. Therefore, the hepatocellular carcinoma immune prognosis score (HCIPS) based on the clinical and pathological characteristics of HCC patients receiving ICIs may have higher prognostic value than classical biomarkers.

## Materials and methods

### Patients

The subjects of this study were 151 HCC patients receiving ICIs at the Harbin Medical University Cancer Hospital from January 2018 to December 2021. To establish hepatocellular carcinoma immune prognosis score (HCIPS), we collected routine blood test indicators from all patients through a medical record system. Incomplete blood parameters and clinical information were the exclusion criteria for this study, and all analyses complied with the Helsinki Declaration and its amendments. Finally, this study received support from the Ethics Committee of the Harbin Medical University Cancer Hospital (Ethical approval number: ALTN-AK105-III-06).

### Data collection and follow-up

The endpoints of this study were progression-free survival (PFS) and overall survival (OS), which were obtained through routine telephone follow-up. The follow-up period was 40 months. For patients who obtained evidence of tumor progression through various imaging examinations, PFS was defined as the period from the first date of treatment to disease progression; The PFS of the patients without evidence of tumor progression was also defined as the period from the first date of treatment to death or the last follow-up. At the same time, OS was defined as the period from the first date of treatment to the last follow-up and death due to all reasons.

### Treatment methods

All patients received over four cycles of combination therapy involving targeted and immune therapy. Out of the total patient cohort, 77 individuals (constituting 51.0%) received intravenous infusions of atezolizumab at a dosage of 1200 mg, along with bevacizumab at 15 mg/kg, administered every three weeks. The remaining 74 patients (making up 49.0% of the group) participated in a clinical trial, where they received intravenous infusions of camrelizumab at a dose of 200 mg (for patients weighing ≥ 50 kg) or 3 mg/kg (for those weighing < 50 kg), administered every two weeks. In addition, they underwent daily oral administration of apatinib at a dose of 250 mg (Clinical Trial Number: CTR20211710).

### Hepatocellular carcinoma immune prognosis score

**Fig. 1:**
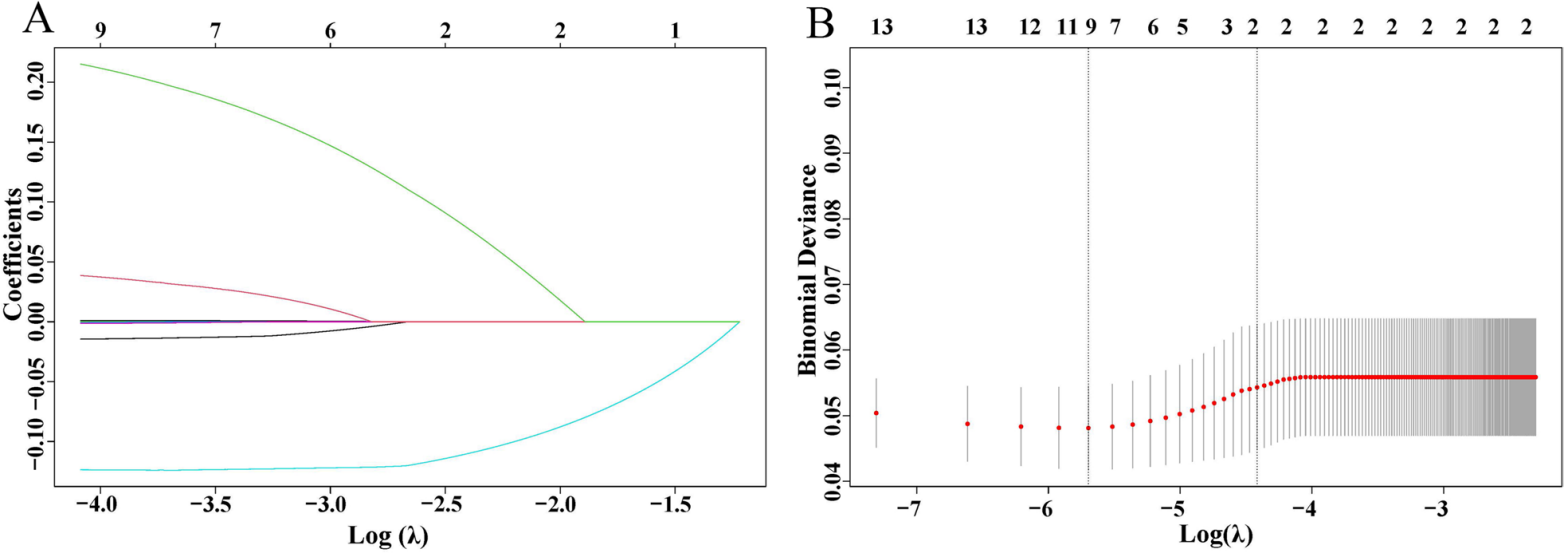
Lasso regression analysis of blood parameters. **A**, **B** Select the optimal lambda interval and blood parameters

All patients had blood samples collected the day before their initial treatment and completed the testing within 2 h. We included all routine blood parameters of patients in the screening process. Univariate and multivariate Cox regression analyses were employed to identify the blood parameters with the most significant impact on OS. To avoid potential information bias during the grouping process, we directly included all blood parameters in the form of continuous variables in Cox’s regression analysis. A significance level of *P* < 0.05 in the univariate analysis was set as the criterion for inclusion in the multivariate analysis. Additionally, to avoid potential multicollinearity, we also subjected all parameters prepared for inclusion in the multivariate analysis to Lasso regression analysis. Univariate analysis revealed that a total of 14 blood parameters were associated with patients’ OS. After incorporating them into the Lasso regression analysis, the regression model obtained the optimal λ value of 0.017 after 572 validations. The results indicated that total bilirubin (TBIL), direct bilirubin (DBIL), uric acid (UA), prealbumin (PALB), and red blood cells (RBC) exhibited multicollinearity and were therefore excluded from the multivariate analysis (Fig. 1).

 After incorporating the remaining blood parameters into the Cox’s multivariate analysis, we found that albumin (ALB) [Hazard ratio (HR) = 0.885, *P* = 0.002] and thrombin time (TT) (HR = 1.253, *P* = 0.022) were independent prognostic factors for OS, with β coefficients of -0.122 and 0.226, respectively (Table 1).

**Table 1:** Univariate and multivariate analysis for blood parameters

	Univariate analysis			Multivariate analysis		
Items	β value	HR (95% CI)	Crude *P*	β value	HR (95% CI)	Adjusted *P*
ALT (U/L)	0.004	1.004(0.999–1.009)	0.086			
AST (U/L)	0.004	1.004(1.001–1.006)	0.003	0.002	1.002(0.998–1.006)	0.402
γ-GGT (U/L)	< 0.001	1.000(0.999–1.002)	0.363			
ALP (U/L)	0.002	1.002(1.000–1.003)	0.012	< 0.001	1.000(0.998–1.002)	0.904
TBIL (μmol/L)	0.004	1.004(1.001–1.006)	0.004			
DBIL (μmol/L)	0.007	1.008(1.003–1.012)	0.002			
IDBIL (μmol/L)	0.008	1.001(1.003–1.014)	0.002	0.025	1.025(0.988–1.064)	0.192
TP (g/L)	-0.042	0.959(0.936–0.982)	0.001	-0.011	0.989(0.954–1.025)	0.544
ALB (g/L)	-0.156	0.856(0.816–0.898)	< 0.001	-0.122	0.885(0.820–0.956)	0.002
GLOB (g/L)	0.005	1.005(0.973–1.039)	0.745			
A/G	0.007	1.007(0.986–1.029)	0.513			
PALB (mg/L)	-0.007	0.993(0.990–0.997)	< 0.001			
Urea (mmol/L)	0.008	1.008(0.969–1.050)	0.686			
CREA (μmol/L)	-0.015	0.985(0.969–1.001)	0.069			
UA (μmol/L)	-0.003	0.997(0.995–1.000)	0.021			
CYS-C (mg/L)	0.202	1.224(0.433–3.465)	0.703			
CO2-CP (mmol/L)	-0.007	0.993(0.925–1.067)	0.855			
LDH (U/L)	0.001	1.001(1.000–1.002)	0.152			
Glu (mmol/L)	0.073	1.075(0.921–1.255)	0.358			
WBC (10^9^/L)	-0.024	0.977(0.887–1.076)	0.633			
NEU (10^9^/L)	-0.032	0.969(0.868–1.081)	0.571			
LYM (10^9^/L)	-0.448	0.639(0.401–1.018)	0.059			
MON (10^9^/L)	0.455	1.576(0.838–2.963)	0.158			
RBC (10^9^/L)	-0.512	0.599(0.406–0.884)	0.010			
HGB (10^9^/L)	-0.015	0.985(0.976–0.994)	0.001	-0.003	0.997(0.975–1.020)	0.816
HCT (10^9^/L)	-0.077	0.926(0.889–0.964)	< 0.001	-0.077	0.926(0.818–1.049)	0.226
PLT (10^9^/L)	-0.002	0.998(0.996–1.001)	0.257			
PT (s)	0.278	1.321(1.152–1.516)	< 0.001	0.073	1.076(0.878–1.319)	0.480
INR	0.006	1.006(0.980–1.032)	0.662			
Fbg (g/L)	0.015	1.015(0.882–1.169)	0.834			
TT (s)	0.287	1.332(1.090–1.627)	0.005	0.226	1.253(1.033–1.521)	0.022

*ALT* Alanine transaminase, *AST* Aspartate aminotransferase, *γ-GGT* γ-glutamyl transferase, *ALP* Alkaline phosphatase, *TBIL* Total bilirubin, *DBIL* Direct bilirubin, *IDBIL* Indirect bilirubin, *GLOB* Globulin, *Urea* Urea nitrogen, *CREA* Creatinine, *UA* uric acid, *CYS-C* Cystatin C, *CO2-CP* CO2 combining power, *LDH* Lactate dehydrogenase, *Glu* Glucose, *WBC* White blood cell, *NEU* Neutrophils, *LYM* Lymphocyte, *MON* Monocyte, *HCT* Hematocrit, *INR* International normalized ratio, *Fbg* Fibrinogen

Due to the findings that ALB and TT were identified as the blood parameters with the most significant impact on OS in this study, we used them to establish HCIPS. The calculation formula for HCIPS was as follows: HCIPS = 0.122 × ALB − 0.226 × TT (s). In addition, to demonstrate that HCIPS had stronger prognostic predictive ability than classical non-invasive biomarkers, we also calculated PNI, SII, and ALI value, their calculation formula was as follows: PNI = albumin (g/L) + 5 × lymphocyte (10^9^/L); SII = platelet (10^9^/L) × neutrophil (10^9^/L) / lymphocyte (10^9^/L); ALI = BMI (Kg/m^2^) × albumin (g/dL) × lymphocyte (10^9^/L) / neutrophil (10^9^/L). All cut-off values and area under the curve (AUC) in this study were calculated using the receiver operating characteristic (ROC) curves based on death.

### Statistical analysis

We performed all statistical analysis and charting by R 4.2.1, GraphPad 8.0, and SPSS 25.0, and two-sided *P* values < 0.05 was defined as having statistical differences. Continuous variables were expressed as mean ± standard deviation (SD) and categorical variables were expressed as the number of patients and percentage (%). The differences in clinical characteristics were compared by the independent-sample t test, Pearson correlation analysis, Chi-square test, or Fisher’s exact test, while survival differences were compared by the Log-rank test. In addition, Cox’s regression analysis was used to find independent prognostic indicators. Finally, the prognostic value of HCIPS was further explored through stratified analysis and the construction of nomograms.

## Results

### Patient characteristics

**Fig. 2:**
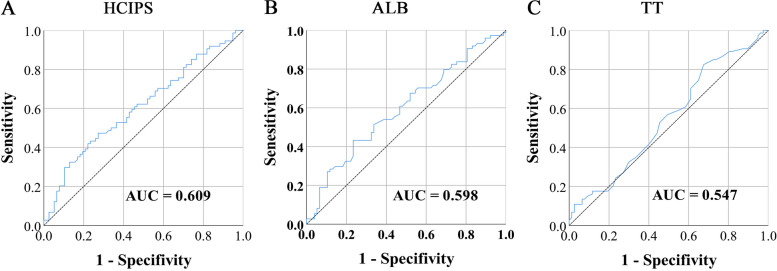
The ROC curves of HCIPS and related markers. **A** The ROC curve of HCIPS; **B** The ROC curve of ALB; **C** The ROC curve of TT

 Out of the 151 patients who were administered ICIs, 124 (82.1%) were men and 27 (17.9%) were women, and their mean age was 57.41 (9.14) years. Due to the high BCLC and TNM stage of the cases in this study, only 51 patients (33.8%) underwent surgery. In addition, we grouped patients based on the median of carcinoembryonic antigen (CEA), alpha-fetoprotein (AFP), and carbohydrate antigen 199 (CA199). The maximum Youden index of HCIPS calculated through ROC was 0.200, with a cut-off value of 0.64 (Fig. 2A). There were 56 cases (37.1%) with HCIPS < 0.64 and 95 cases (62.9%) with HCIPS ≥ 0.64. The results showed a significant correlation between low HCIPS and several unfavorable factors, including non-surgery, BCLC stage C, and TNM stage III+IV (all *P* < 0.05). In addition, Fisher’s exact test found HCIPS was also related to tumor size, which all reflected the prognostic value of HCIPS to a certain extent (*P* = 0.020) (Table 2).

**Table 2:** Patient characteristics

	HCIPS		*P*
Items (%)	< 0.64 (*n* = 56)	≥ 0.64 (*n* = 95)	
Sex			0.995
Male	46(82.1)	78(82.1)	
Female	10(17.9)	17(17.9)	
Age (years), mean (SD)	57.70(10.23)	57.24(8.49)	0.769
BMI (Kg/m^2^), mean (SD)	22.73(3.75)	23.70(3.42)	0.106
Smoking			0.532
Yes	10(17.9)	21(22.1)	
No	46(82.1)	74(77.9)	
Drinking			0.981
Yes	7(12.5)	12(12.6)	
No	49(87.5)	83(87.4)	
ABO blood type			0.788
A	13(23.2)	29(30.5)	
B	18(32.1)	27(28.4)	
AB	8(14.3)	14(14.7)	
O	17(30.4)	25(26.3)	
Surgery			0.001
Yes	10(17.9)	41(43.2)	
No	46(82.1)	54(56.8)	
Tumor number			0.495
Single	21(37.5)	41(43.2)	
Multiple	35(62.5)	54(56.8)	
Tumor size			0.02
< 5 cm	5(8.9)	23(24.2)	
≥ 5 cm	51(91.1)	72(75.8)	
Liver cirrhosis			0.395
Yes	19(33.9)	26(27.4)	
No	37(66.1)	69(72.6)	
BCLC stage			0.028
A + B	18(32.1)	48(50.5)	
C	38(67.9)	47(49.5)	
TNM stage			0.002
I + II	13(23.2)	46(48.4)	
III + IV	43(76.8)	49(51.6)	
CEA			0.199
< 2.38 ng/mL	24(42.9)	51(53.7)	
≥ 2.38 ng/mL	32(57.1)	44(46.3)	
AFP			0.343
< 151.4 ng/mL	25(44.6)	50(52.6)	
≥ 151.4 ng/mL	31(55.4)	45(47.4)	
CA199			0.246
< 22.64 U/mL	24(42.9)	50(52.6)	
≥ 22.64 U/mL	32(57.1)	45(47.4)	

**Fig. 3:**
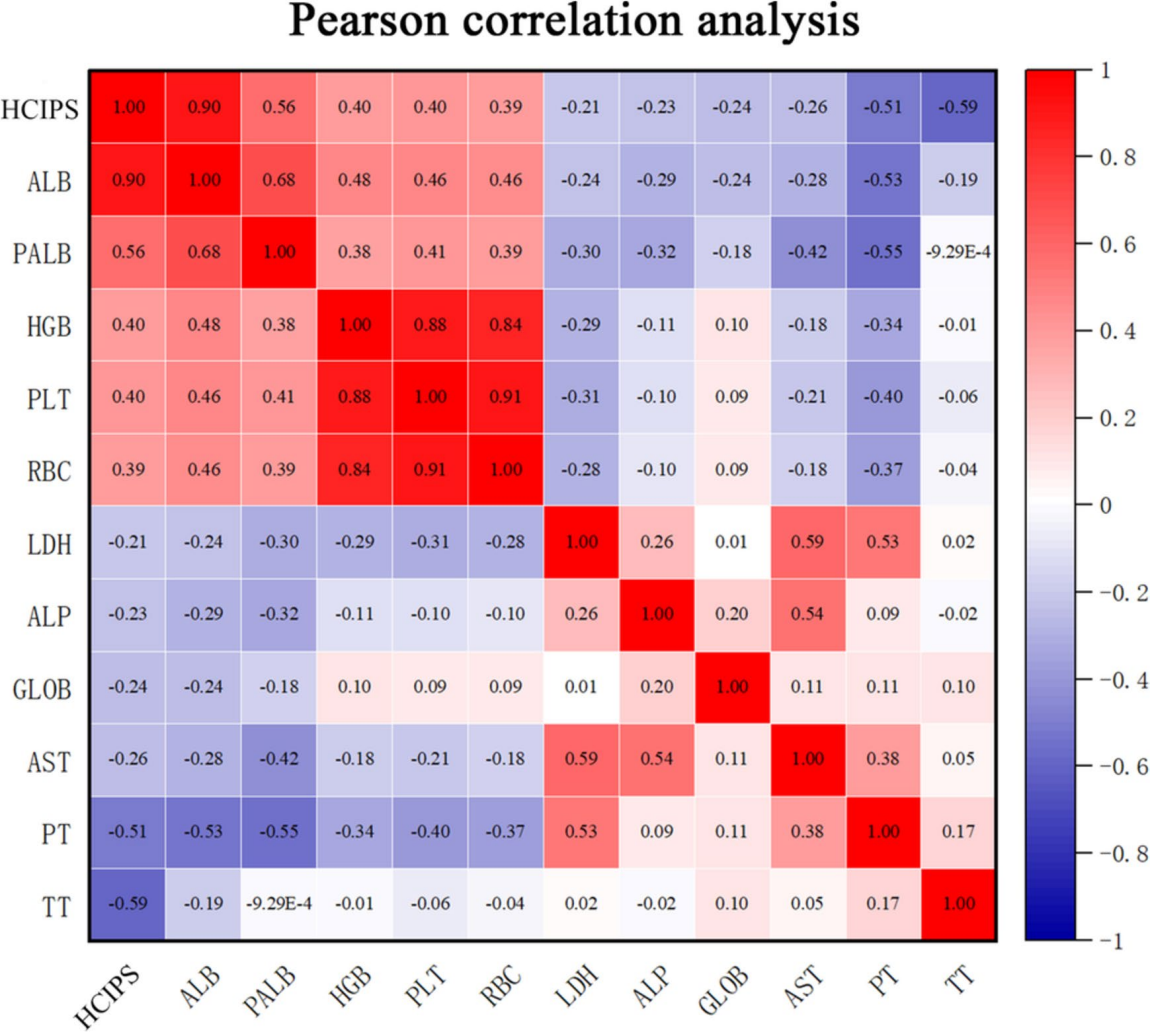
Pearson’s analysis of HCIPS. LDH: lactate dehydrogenase; ALP, alkaline phosphatase; GLOB: globulin; AST: Aspartate aminotransferase

 The correlation analysis of blood parameters also revealed that HCIPS was associated with a wide range of blood parameters, as detailed in Table 3. We also performed Pearson’s analysis of HCIPS and other blood parameters and found that ALB, PALB, hemoglobin (HGB), platelet (PLT), and red blood cell (RBC) were significantly positively correlated with HCIPS (R > 0.3, *P* < 0.05), while prothrombin time (PT) and TT were significantly negatively correlated with HCIPS (R <-0.3, *P* < 0.05) (Fig. 3).

**Table 3:** Patient blood parameters

	HCIPS		*P*
Item, Mean (SD)	< 0.64 (*n* = 56)	≥ 0.64 (*n* = 95)	
ALT (U/L)	54.88(58.55)	38.06(30.02)	0.022
AST (U/L)	101.61(103.07)	58.40(58.69)	0.005
γ-GGT (U/L)	230.66(224.93)	137.39(152.85)	0.007
ALP (U/L)	221.68(172.17)	139.00(98.17)	0.002
TBIL (μmol/L)	51.55(91.10)	21.50(13.43)	0.017
DBIL (μmol/L)	21.96(47.20)	5.95(6.01)	0.014
IDBIL (μmol/L)	30.98(44.09)	15.68(7.90)	0.013
TP (g/L)	67.96(7.38)	82.35(7.51)	0.168
ALB (g/L)	32.13(3.87)	40.95(3.26)	< 0.001
GLOB (g/L)	35.83(7.70)	33.71(7.49)	0.099
PALB (mg/L)	105.29(54.16)	181.08(70.15)	< 0.001
Urea (mmol/L)	5.84(2.83)	6.46(6.00)	0.471
CREA (μmol/L)	68.40(13.49)	76.26(15.28)	0.002
UA (μmol/L)	292.54(110.65)	318.01(103.86)	0.157
CYS-C (mg/L)	0.95(0.24)	0.91(0.22)	0.223
CO2-CP (mmol/L)	26.05(3.63)	25.65(2.89)	0.454
LDH (U/L)	367.36(88.93)	228.91(83.30)	0.011
Glu (mmol/L)	5.33(1.51)	5.77(1.56)	0.089
WBC (10^9^/L)	6.69(3.25)	6.35(2.21)	0.449
LYM (10^9^/L)	1.26(0.61)	1.37(0.51)	0.219
NEU (10^9^/L)	4.81(2.81)	4.34(2.03)	0.274
MON (10^9^/L)	0.57(0.36)	0.47(0.24)	0.066
RBC (10^9^/L)	4.13(0.77)	4.63(0.62)	< 0.001
HGB (10^9^/L)	128.29(23.06)	144.05(22.42)	< 0.001
HCT (10^9^/L)	38.68(6.73)	43.32(6.03)	< 0.001
PLT (10^9^/L)	177.27(130.36)	169.57(69.31)	0.636
PT (s)	13.39(2.16)	12.09(1.02)	< 0.001
INR	2.37(9.03)	2.05(7.45)	0.815
Fbg (g/L)	3.11(1.35)	3.46(1.83)	0.225
TT (s)	17.52(1.51)	16.49(1.52)	< 0.001

*ALT* Alanine transaminase, *AST* Aspartate aminotransferase, *γ-GGT* γ-glutamyl transferase, *ALP* Alkaline phosphatase, *TBIL* Total bilirubin, *DBIL* Direct bilirubin, *IDBIL* Indirect bilirubin, *GLOB* Globulin, *Urea* Urea nitrogen, *CREA* Creatinine, *UA* uric acid, *CYS-C* Cystatin C, *CO2-CP* CO2 combining power, *LDH* Lactate dehydrogenase, *Glu* Glucose, *WBC* White blood cell, *NEU* Neutrophils, *LYM* Lymphocyte, *MON* Monocyte, *HCT* Hematocrit, *INR* International normalized ratio, *Fbg* Fibrinogen

### Prognostic value of hepatocellular carcinoma immune prognosis score

 We calculated the AUC of all clinical data and blood parameters using ROC curves. The results revealed that HCIPS had a significantly higher AUC than ALB and TT alone, and was, in fact, the factor with the highest AUC in this study, underscoring the tremendous prognostic value of HCIPS. The detailed AUC values of significant prognostic factors were shown in Table 4.

**Table 4:** Area under curve for different biomarkers

Items	AUC	95% CI
ALB	0.598	0.507–0.688
TT	0.547	0.455–0.639
HCIPS	0.609	0.519–0.699
BMI	0.565	0.473–0.657
BCLC stage	0.584	0.493–0.675
TNM stage	0.565	0.474–0.657
Surgery	0.579	0.488–0.671
Tumor size	0.576	0.485–0.667
Tumor number	0.571	0.480–0.663
PNI	0.566	0.475–0.658
SII	0.564	0.472–0.655
ALI	0.598	0.507–0.688
AST	0.565	0.473–0.658
TP	0.580	0.489–0.671
UA	0.566	0.475–0.658
LYM	0.582	0.490–0.673
MON	0.592	0.501–0.683

*AST* Aspartate aminotransferase, *UA* Uric acid, *LYM* Lymphocyte, *MON* Monocyte

### Survival analysis of hepatocellular carcinoma immune prognosis score

**Fig. 4:**
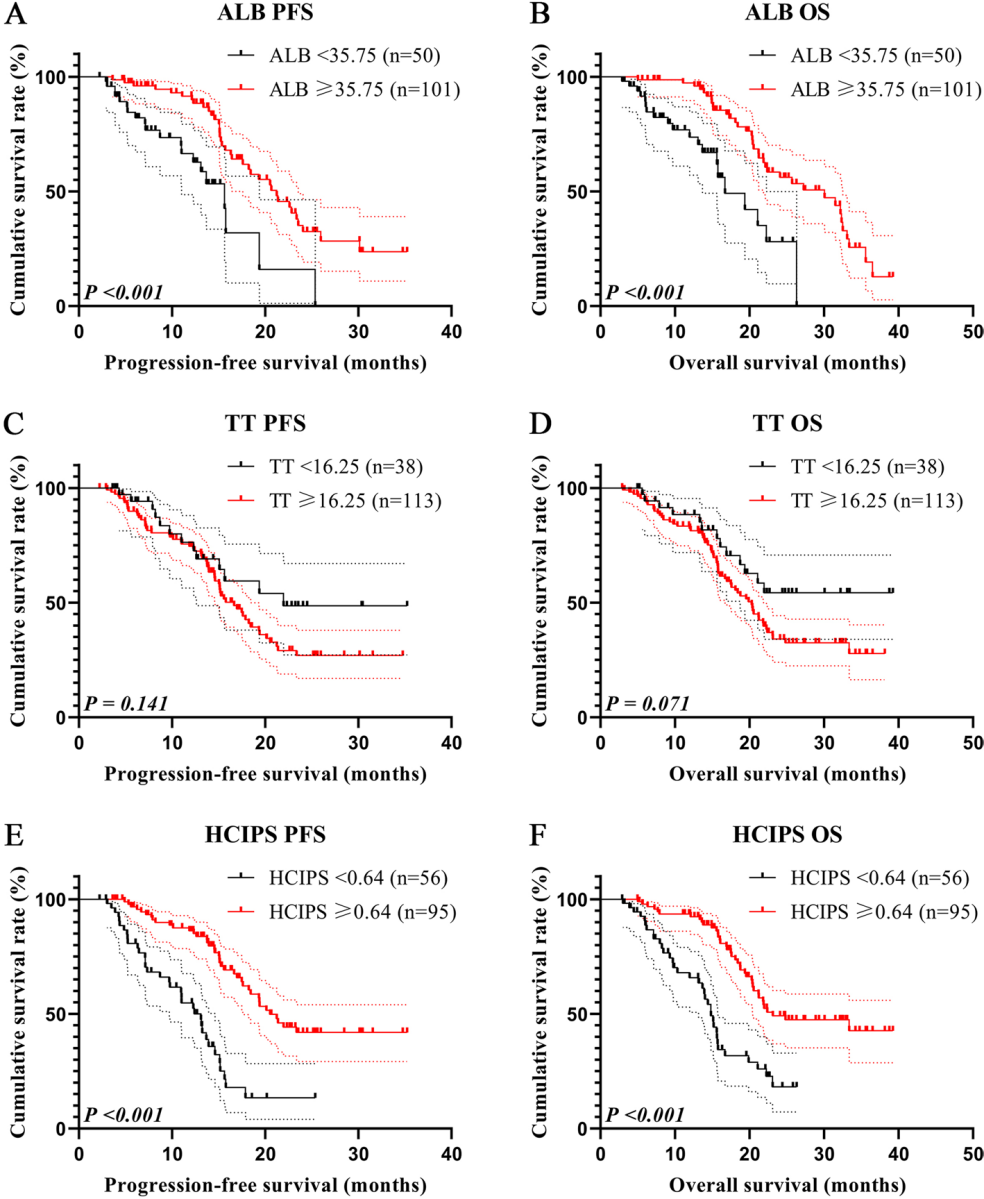
Survival curve of HCIPS and related markers for PFS and OS. **A** Survival curve of ALB for PFS; **B** Survival curve of ALB for OS; **C** Survival curve of TT for PFS; **D** Survival curve of TT for OS; **E** Survival curve of HCIPS for PFS; **F** Survival curve of HCIPS for OS

We conducted survival analyses on the grouped HCIPS and its constituent indicators. The maximum Youden index of ALB was 0.199, with a cut-off value of 35.75 g/L (Fig. 2B). There were 50 cases with ALB < 35.75 g/L and 101 cases with ALB ≥ < 35.75 g/L, patients with low ALB had shorter PFS (11.02 months vs. 20.53 months, *P* < 0.001) and OS (14.40 months vs. 23.13 months, *P* < 0.001) (Fig. 4A, B). In addition, the maximum Youden index and the cut-off value of TT were 0.149 and 16.25 s (Fig. 2C). 38 patients were enrolled in the low TT group and 113 patients were enrolled in the high TT group. The longer TT was also related to shorter PFS and OS (28.29 months vs. 16.66 months, *P* = 141 and not reached vs. 20.30 months, *P* = 0.071) (Fig. 4C, D). Finally, patients with low HCIPS had significantly poorer PFS (13.10 months vs. 21.63 months, *P* < 0.001) and OS (14.83 months vs. 25.43 months, *P* < 0.001) (Fig. 4E, F).

### Univariate and multivariate cox’s regression analysis

 To further explore the prognostic value of HCIPS, we conducted Cox’s regression analysis with the clinical data of patients. We found that ALB, TT, HCIPS, surgery, tumor number, tumor size, liver cirrhosis, BCLC stage, and TNM stage were related to the PFS (all *P* < 0.05). At the same time, HCIPS (*P* = 0.001) and TNM stage (*P* = 0.007) were both found to be the independent prognostic markers for PFS (Table 5). In addition, OS was related to sex, ALB, HCIPS, surgery, tumor number, tumor size, BCLC stage, and TNM stage (all *P* < 0.05), while HCIPS, tumor size, and TNM stage were also the independent prognostic markers for OS (Table 6).

**Table 5:** Univariate and multivariate analysis for PFS

			PFS			
	Univariate analysis			Multivariate analysis		
Items	HR	95 %CI	*Crude P*	HR	95 %CI	*Adjusted P*
Sex (Male vs Female)						
Male	Ref					
Female	1.671	0.990–2.822	0.055			
Age	0.999	0.974–1.025	0.953			
BMI	0.934	0.872–1.001	0.054			
ALB						
< 35.75 g/L	Ref					
≥ 35.75 g/L	0.233	0.143–0.381	< 0.001			
TT						
< 16.25 s	Ref					
≥ 16.25 s	1.564	0.858–2.851	0.144			
HCIPS						
< 0.639	Ref			Ref		
≥ 0.639	0.268	0.165–0.434	< 0.001	0.417	0.251–0.693	0.001
CEA						
< 2.38 U/mL	Ref					
≥ 2.38 U/mL	1.299	0.822–2.053	0.262			
AFP						
< 151.4 U/mL	Ref					
≥ 151.4 U/mL	1.275	0.807–2.013	0.297			
CA199						
< 22.64 U/mL	Ref					
≥ 22.64 U/mL	1.231	0.779–1.945	0.373			
Surgery						
Yes	Ref			Ref		
No	2.725	1.602–4.635	< 0.001	1.259	0.669–2.371	0.475
Tumor number						
Single	Ref			Ref		
Multiple	1.763	1.086–2.861	0.022	1.375	0.833–2.270	0.213
Tumor size						
< 5 cm	Ref			Ref		
≥ 5 cm	3.227	1.540–6.761	0.002	2.054	0.913–4.619	0.082
Liver cirrhosis						
Yes	Ref			Ref		
No	0.578	0.356–0.940	0.027	0.714	0.436–1.170	0.181
BCLC						
A + B	Ref			Ref		
C	2.974	1.823–4.853	< 0.001	1.601	0.879–2.915	0.124
TNM stage						
I + II	Ref			Ref		
III + IV	3.363	2.031–5.570	< 0.001	2.289	1.259–4.161	0.007

**Table 6:** Univariate and multivariate analysis for OS

			OS			
	Univariate analysis			Multivariate analysis		
Items	HR	95 % CI	*Crude P*	HR	95 % CI	*Adjusted P*
Sex (Male vs Female)						
Male	Ref			Ref		
Female	1.851	1.097–3.123	0.021	1.083	0.615–1.907	0.782
Age	0.999	0.974–1.024	0.923			
BMI	0.952	0.887–1.021	0.17			
ALB						
< 35.75 g/L	Ref					
≥ 35.75 g/L	0.263	0.163–0.423	< 0.001			
TT						
< 16.25 s	Ref					
≥ 16.25 s	1.724	0.947–3.139	0.075			
HCIPS						
< 0.639	Ref			Ref		
≥ 0.639	0.334	0.209–0.533	< 0.001	0.507	0.309–0.833	0.007
CEA						
< 2.38 U/mL	Ref					
≥ 2.38 U/mL	1.381	0.874–2.184	0.167			
AFP						
< 151.4 U/mL	Ref					
≥ 151.4 U/mL	1.384	0.875–2.188	0.164			
CA199						
< 22.64 U/mL	Ref					
≥ 22.64 U/Ml	1.122	0.711–1.771	0.621			
Surgery						
Yes	Ref			Ref		
No	2.986	1.751–5.093	< 0.001	1.527	0.838–2.784	0.167
Tumor number						
Single	Ref			Ref		
Multiple	1.811	1.117–2.937	0.016	1.556	0.926–2.615	0.095
Tumor size						
< 5	Ref			Ref		
≥ 5	3.364	1.606–7.047	0.001	2.328	1.032–5.249	0.042
Liver cirrhosis						
Yes	Ref					
No	0.632	0.389–1.025	0.063			
BCLC						
A + B	Ref			Ref		
C	3.47	2.114–5.693	< 0.001	1.693	0.923–3.106	0.089
TNM stage						
I + II	Ref			Ref		
III + IV	3.682	2.224–6.097	< 0.001	2.802	1.504–5.220	0.001

### Stratified analyses by potential effect modifiers

**Fig. 5:**
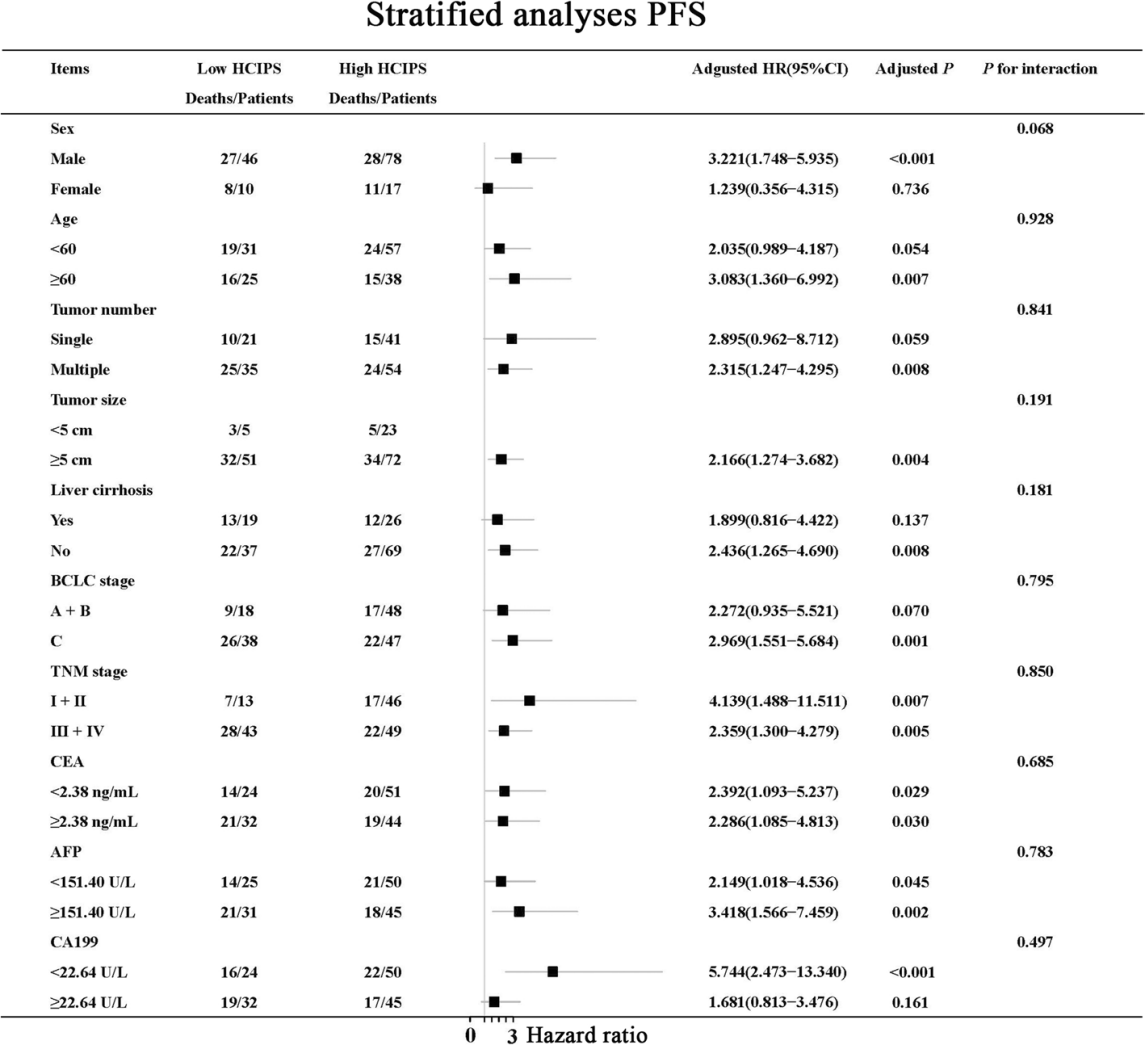
Stratified analyses of HCIPS for PFS

Since HCIPS was an independent prognostic indicator for PFS and OS, we conducted the stratified analyses for HCIPS based on the multivariate analysis parameters. We observed a significant correlation between HCIPS and PFS in male patients, those aged ≥ 60 years, those with tumor size ≥ 5 cm, those without liver cirrhosis, those with BCLC stage C, all TNM stages, all CEA groups, all AFP groups, and those with CA199 < 22.64 U/L (all *P* < 0.05) (Fig. 5).

**Fig. 6:**
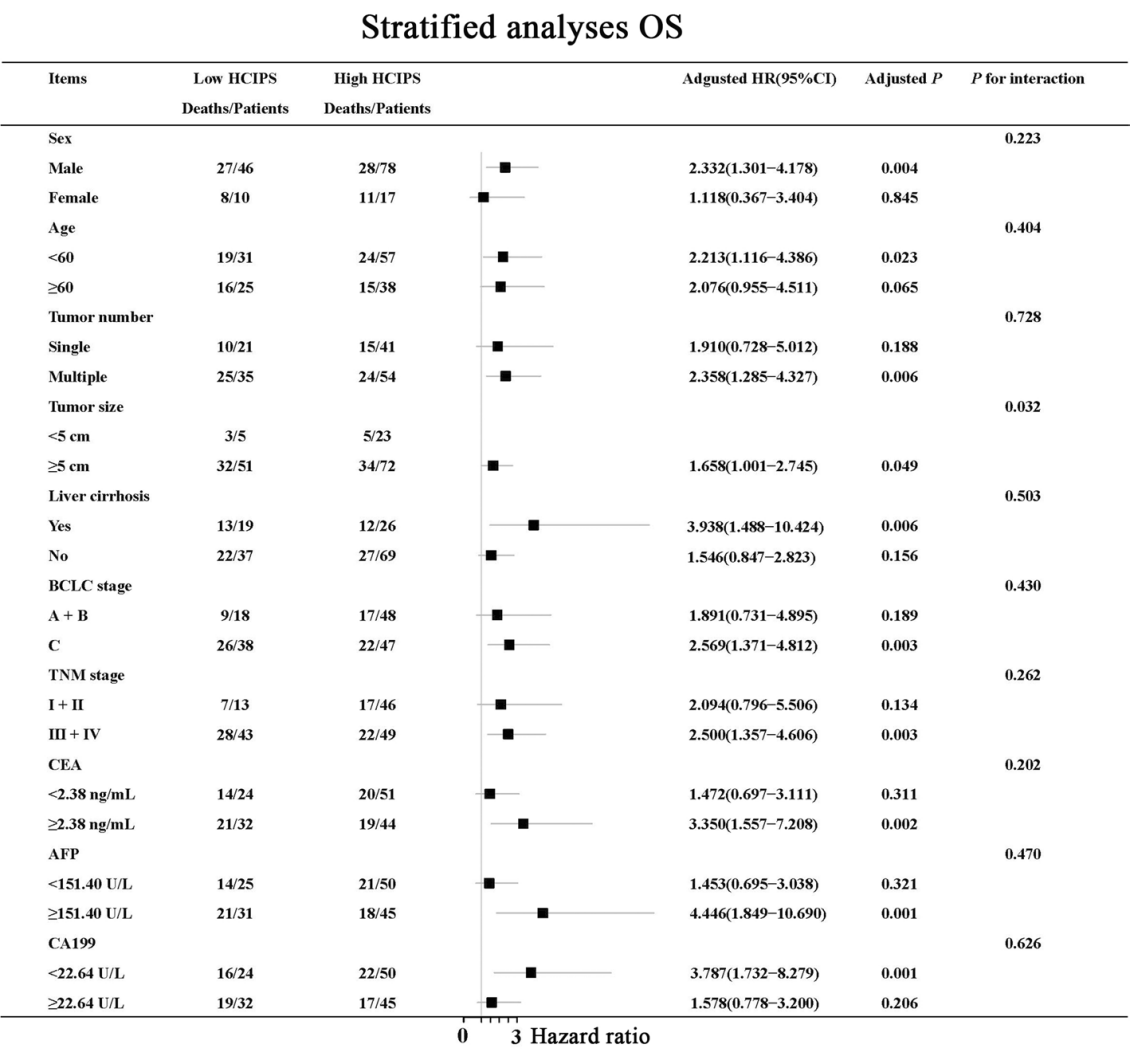
Stratified analyses of HCIPS for OS

At the same time, we found that HCIPS was closely related to OS in male patients, those aged < 60 years, those with tumor size ≥ 5 cm, those with liver cirrhosis, those with BCLC stage C, those with TNM stage III+IV, those with CEA ≥ 2.38 U/L, those with AFP ≥ 151.40 U/L, and those with CA199 < 22.64 U/L (all *P* < 0.05) (Fig. 6). It was worth noting that we found a significant correlation between the prognostic value of HCIPS and tumor size ≥ 5 cm (*P* for interaction = 0.032).

### Nomograms predicted 1-year survival probability

**Fig. 7:**
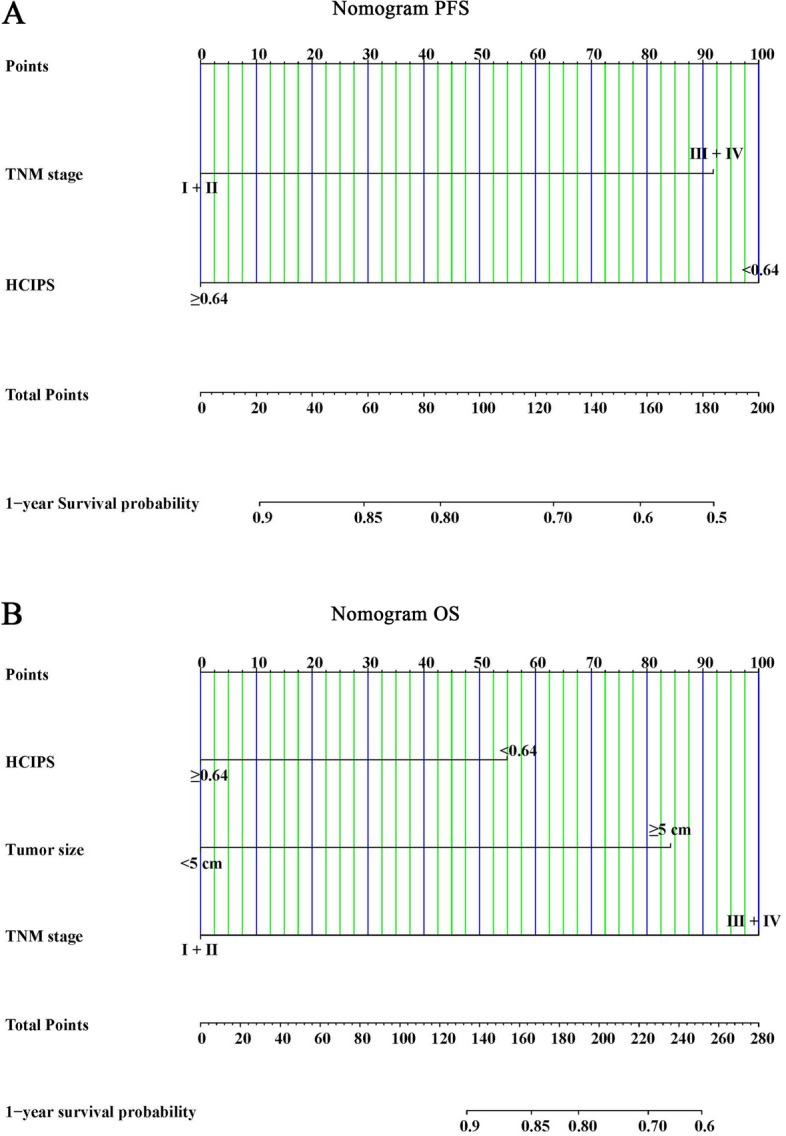
Nomograms predicted survival probability for PFS and OS. **A** Nomogram for PFS; **B** Nomogram for OS

**Fig. 8:**
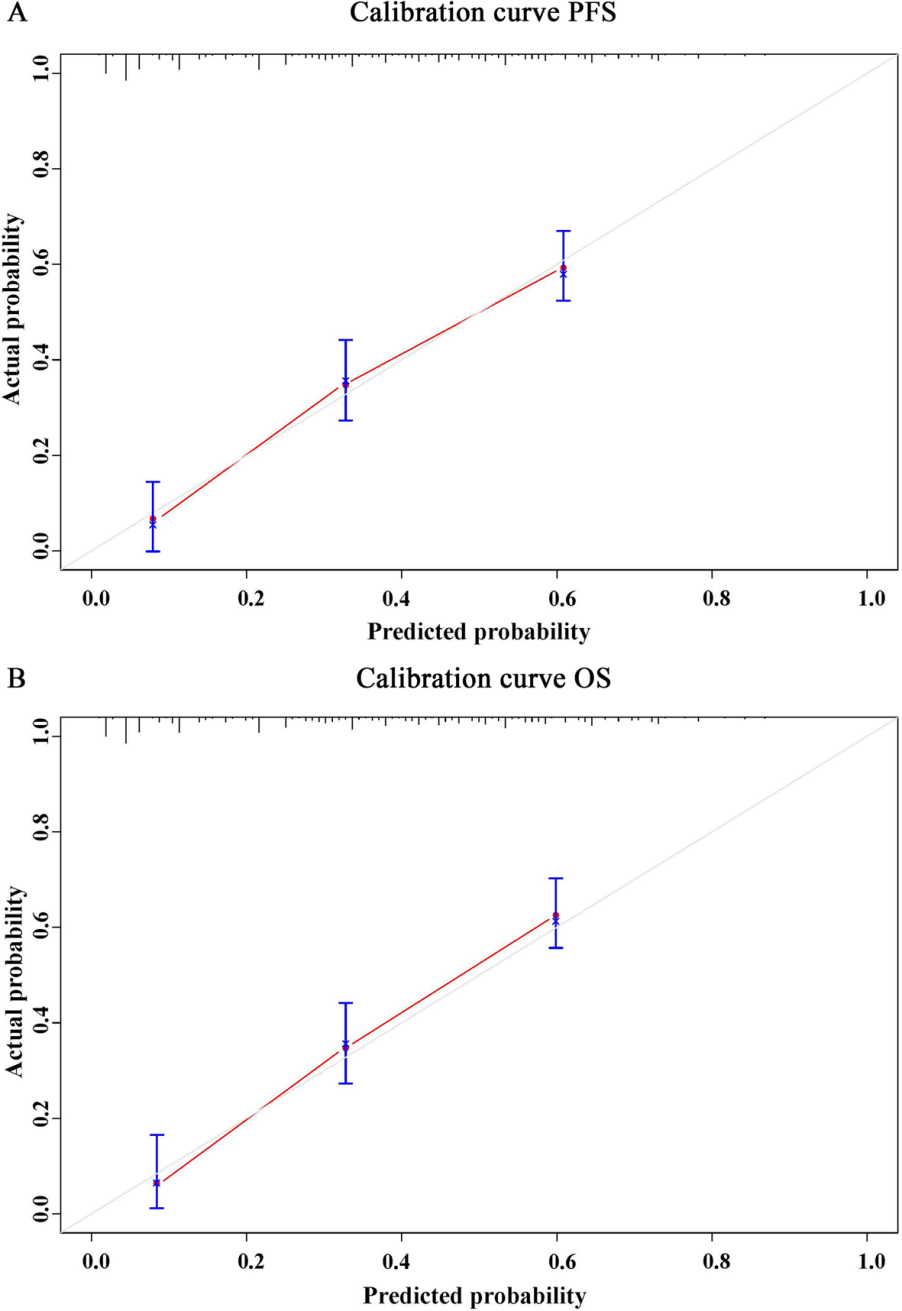
The calibration curves of nomograms. **A** Calibration curve of nomogram for PFS; **B** Calibration curve of nomogram for OS

**Fig. 9:**
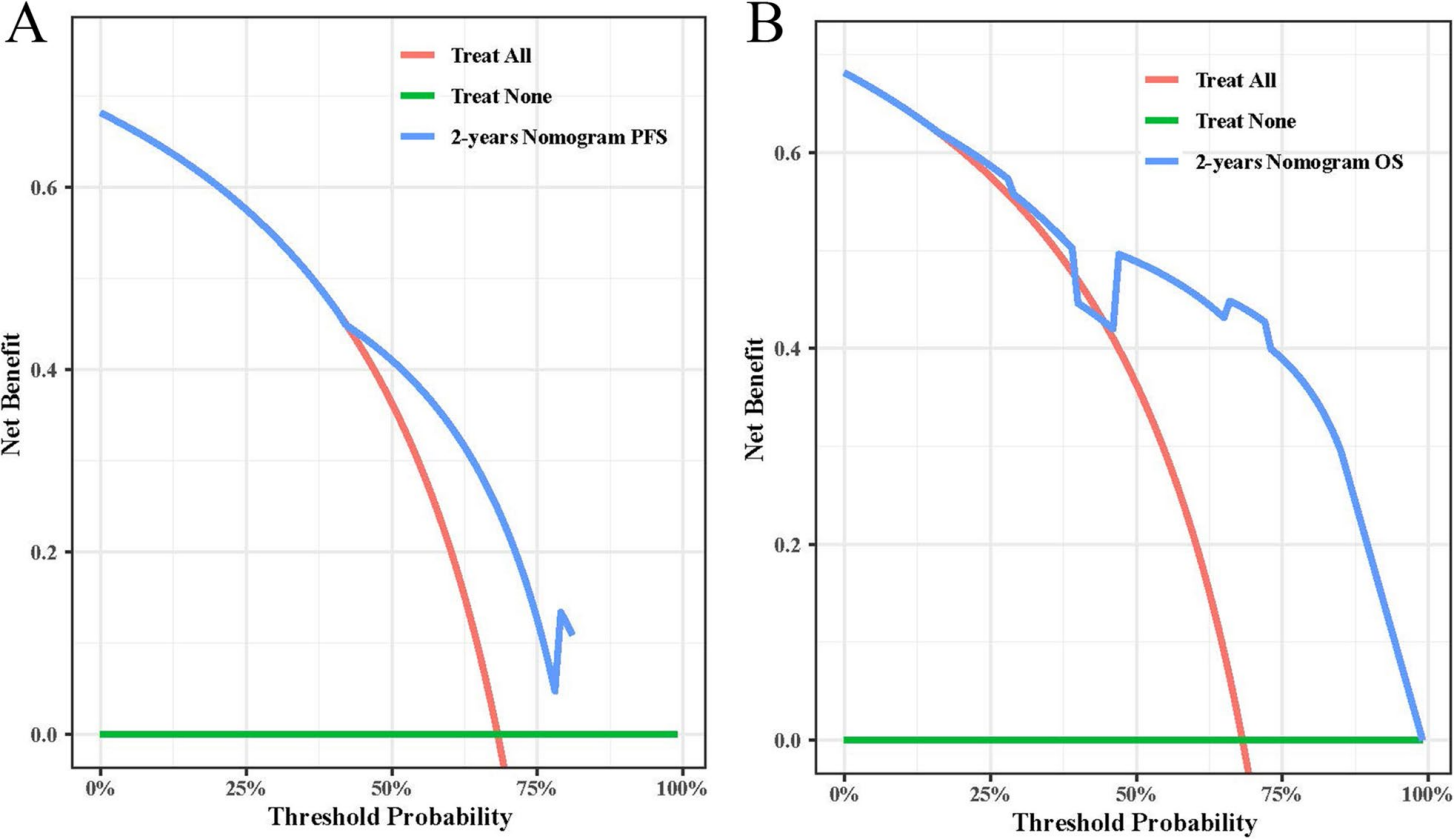
The DCA curves for PFS and OS. **A** The DCA curve of nomogram for PFS; **B** The DCA curve of nomogram for OS

Finally, we drew nomograms for PFS and OS based on the results of multivariate analysis, with the C-index and 95%CI of 0.730(0.680–0.779) and 0.758(0.711–0.804) (Fig. 7A, B). In Addition, due to the limitation of the number of patients, we conducted the bootstrap correction for nomograms and drew the calibration curves. They all showed high predictive effectiveness of nomographs (Fig. 8A, B). We also plotted Clinical Decision Analysis (DCA) curves to further validate the predictive accuracy of the nomograms. The DCA curves also demonstrated the powerful predictive ability of nomograms (Fig. 9A, B).

## Discussion

Due to the low resection rate of HCC, biomarkers such as PD-L1 that predicted the efficacy of immunotherapy were difficult to obtain for many patients. For them, non-invasive biomarkers might have a higher value. Although many classic inflammatory and nutritional markers have been found to identify patients who benefit from ICIs, most of them were not exclusive markers of HCC and ICIs. Therefore, this study established a new immune prognosis score based on clinical data of HCC patients receiving ICIs, providing a new direction for searching for ICIs related biomarkers.

So far, many studies have been conducted on non-invasive biomarkers related to ICIs. In a study on gastric cancer conducted in 2022, Sun et al. analyzed approximately 90 patients who received ICIs and found that PNI was significantly correlated with prognosis and was an independent prognostic factor for patients receiving ICIs [17]. Another study on lung cancer also obtained similar results. In 2019, Shoji et al. collected clinical data from 102 non-small cell lung cancer (NSCLC) patients who received ICIs and found a significant correlation between PNI and patient response to ICIs treatment [25]. SII reflected the inflammatory state of patients, Chen et al. collected 139 gastric cancer patients in 2021 and analyzed the application value of SII in ICIs patients. After analysis, they found that SII was also closely related to the prognosis of patients with ICIs [26]. Other studies had also confirmed the prognostic role of various classic non-invasive biomarkers in ICIs [18, 27, 28]. The biomarkers for HCC immunotherapy were also constantly being studied. Zhang et al. found another prognostic marker for HCC patients receiving ICIs through their research on C-reactive protein (CRP). They collected 101 HCC patients in 2022 and analyzed the impact of CRP and CRP combined with AFP on prognosis. The results showed that both CRP and combined indicators showed strong predictive ability for prognosis [29]. Another study successfully predicted the efficacy of ICIs in advanced HCC patients by establishing a predictive model based on the imaging features of ICIs patients [30]. People were also constantly establishing new biomarkers for immunotherapy. Mezquita and his colleagues established the lung Immune Prognostic Index (LIPI) by analyzing 466 NSCLC patients receiving ICIs from 8 centers and they found a significant correlation between LIPI and clinical outcomes in ICIs patients, but not in chemotherapy patients [31]. In 2021, Cao et al. calculated the β coefficient of oxidative stress indicators using Cox’s regression analysis and established a new oxidative stress biomarker. Their results of analyses also successfully demonstrated its prognostic value in colorectal cancer patients undergoing surgery [32]. The new immune prognosis score of this study was also based on the β coefficient and showed a high prognostic value.

After conducting Cox’s regression analysis on all blood parameters, we identified ALB and TT as significant factors affecting OS and established HCIPS. The ROC curve based on death demonstrated the significant advantage of HCIPS in predicting the patient prognosis (AUC = 0.609). Survival analysis of HCIPS found that grouped HCIPS was related to clinical outcomes of HCC, with low HCIPS patients having shorter PFS and OS. In addition, the multivariate analysis also found that HCIPS was a powerful independent prognostic factor. The results of stratified analysis further demonstrated the prognostic value of HCIPS in different patients. It was worth noting that we have found an interaction between HCIPS and tumor size ≥ 5 cm in predicting the patient prognosis (*P* for interaction = 0.032). Finally, the C-index and calibration curve also demonstrated the accuracy of the nomograms containing HCIPS.

HCIPS was composed of ALB and TT, which were important indicators of liver function. ALB reflected the nutritional status of patients, which was closely related to tumor progression [33]. Numerous studies have confirmed the prognostic value of ALB in different tumors, especially hepatocellular carcinoma [34–36]. In addition, the liver was the site of serum protein synthesis, and impaired liver function could lead to a decrease in ALB levels [37]. In this study, although total protein (TP), ALB, and PALB were all related to the prognosis of patients in the preliminary analysis, the longer half-life period of ALB made it more stable in the blood. In addition, the nutritional status of HCC patients was less affected than other digestive cancers, making ALB more reflective of long-term liver damage. Since most components of the coagulation system were synthesized in the liver, the coagulation state of patients was also closely related to liver function [38]. Many studies have found that coagulation status could reflect the clinical outcomes of various cancers, and PT and TT were both prognostic indicators for HCC patients in this study [39–41]. Although PT was the most important indicator for detecting the coagulation status of patients, it was influenced by various factors such as liver synthesis function and inflammatory factors [42]. TT was the time at which fibrinogen was converted into fibrin after the addition of thrombin, and the prolongation of TT to a certain extent reflected the level and state of fibrinogen [43]. The decrease in fibrinogen levels also indicated liver synthesis dysfunction, reflecting long-term damage to liver function [44]. Therefore, TT showed higher prognostic value in multivariate analysis. In addition, cancer patients were in a state of oxidative stress due to tissue damage and the role of inflammatory factors [45, 46]. Oxidative stress could cause the denaturation of albumin and fibrinogen, leading to a rapid decrease in serum albumin levels and a significant prolongation of TT [47]. There was also a close relationship between oxidative stress and immune function. On the one hand, oxidative stress could lead to abnormal immune cell function. On the other hand, the activation state of immune cells could also cause oxidative stress reactions, increasing the degree and duration of oxidative stress [48]. This affected the efficacy of ICIs that rely on normal immune function. Therefore, HCIPS was composed of ALB and TT, which could accurately predict the prognosis of HCC patients receiving ICIs [7].

This study had some inevitable limitations. Firstly, ICIs were still not a conventional treatment for HCC, resulting in a small number of patients in this study. The prognostic value of HCIPS could not be further validated in extensive data. Secondly, this was a single-center retrospective study that could not eliminate potential information bias. Finally, due to patient limitations, this study failed to compare the predictive ability of HCIPS among HCC patients receiving other treatment options. The conclusions of this study needed to be continuously tested in subsequent studies, especially prospective studies with larger sample sizes.

## Conclusions

As a new score established based on HCC patients receiving ICIs, HCIPS was significantly correlated with clinical outcomes in patients with ICIs and might serve as a new biomarker to predict HCC patients who cloud benefit from ICIs.

## Data Availability

The authors promise to provide the original data supporting this study without reservation (Provided by Zhaowei Qu, Email: quzhaowei@hrbmu.edu.cn).
